# Impact of sex on clinical management and mortality in community-onset *Staphylococcus aureus* bacteraemia: a retrospective study

**DOI:** 10.1186/s12879-026-12831-8

**Published:** 2026-03-27

**Authors:** Sonia Matharu, Eugene Athan, Carly L. Botheras

**Affiliations:** 1 Centre for Innovation in Infectious Disease and Immunology Research (CIIDIR), School of Medicine, Deakin University, Geelong, VIC Australia; 2 Institute of Mental and Physical Health and Clinical Translation (IMPACT), School of Medicine, Deakin University, Geelong, VIC Australia; 3 Department of Infectious Disease, University Hospital Geelong, Barwon Health, Geelong, VIC Australia

**Keywords:** *Staphylococcus aureus* bacteraemia (SAB), Community-onset infections, Sex differences, Clinical management, Transoesophageal echocardiography (TOE), 30-day mortality

## Abstract

**Introduction:**

*Staphylococcus aureus* bacteraemia is a severe infection with potential sex-based disparities. While males have higher infection rates, females may experience poorer clinical outcomes. This study examines sex-based differences in the clinical characteristics, management approaches, and outcomes of community-onset *S. aureus* bacteraemia (COSAB) within a single-centre cohort.

**Methods:**

We conducted a single-centre retrospective study of 279 COSAB cases (2015–2020) at University Hospital Geelong, Australia. Clinical characteristics, diagnostic testing, and 30-day all-cause mortality were compared by sex using univariable binomial logistic regression models.

**Results:**

Analysis of 279 COSAB cases (58.4% males, 41.6% females) showed a higher, though not statistically significant association between 30-day mortality rate in females (21.6%) compared to males (17.2%) (*p* = 0.36). There was no significant difference of age between the sexes (*p* = 0.38) or Charlson Age Comorbidity Index (*p* = 0.71). Males had longer median hospital stay (26 days, IQR: 33.25 compared to females (18 days, IQR: 34). Transoesophageal echocardiography (TOE) was performed less frequently in females than males (OR: 0.58, 95% CI 0.35–0.97; *p* = 0.04), despite comparable rates of infective endocarditis. Females were also associated with a methicillin-susceptible *Staphylococcus aureus* (MSSA) infection (OR: 2.10, 95% CI: 1.13–3.97, *p* = 0.02).

**Conclusion:**

This study identified sex-based differences in the management of COSAB, particularly the lower frequency of TOE performed in females. While there was no significant difference in 30-day mortality between sexes in this underpowered study, these disparities warrant further investigation. Understanding the underlying causes of these differences could help improve clinical practices and ensure equitable management for all patients.

## Introduction

Infectious diseases remain a leading cause of morbidity and mortality worldwide. *Staphylococcus aureus (S. aureus)*, a Gram-positive bacterium, can cause severe infections including bacteraemia (SAB), leading to complications such as sepsis and infective endocarditis [1, 2]. In 2019, *S. aureus* infections were responsible for over one million deaths globally [3].

SAB can be both hospital-acquired or community-onset, with community-onset *Staphylococcus aureus* bacteraemia (COSAB) increasingly recognised as the predominant source of infections [4]. In a global meta-analysis of 341 studies involving 536,791 patients, the 30-day mortality rate for SAB was reported as 18.1% [5]. Notably, sex disparities have been reported in SAB outcomes, with males showing higher incidence rates [6–9], yet emerging evidence suggests that females may experience worse clinical outcomes, including higher mortality [7–9]. This highlights the need for further investigation into how sex influences SAB outcomes worldwide.

In Australia, the incidence of SAB has remained stable at approximately 10 cases per 100,000 people annually [4, 10], reflecting the global trend of overall infection rates [5]. COSAB now accounts for 77.5% of all SAB cases [4]. Notably, the 30-day all-cause mortality rate for COSAB has increased from 12.8% in 2020 to 17.0% in 2022 [4]. Alongside these trends, sex-based disparities have been observed in diagnostic testing practices, with evidence suggesting that females may experience delays in diagnosis due to differences in clinical management [11–13].

While sex disparities in SAB outcomes are increasingly recognised, the mechanisms underlying these differences remain poorly understood. These disparities may be influenced by biological factors such as immune response, hormonal regulation, genetics, as well as non-biological factors such as variations in healthcare access and clinical decision-making [14–16]. One area where such differences have been observed is in the use of transoesophageal echocardiography (TOE), a key diagnostic tool for identifying infective endocarditis (IE), a serious complication of SAB. Studies suggest that females are less likely to receive TOE, potentially contributing to delayed diagnosis and poorer outcomes, including mortality [12, 17]. However, sex-based differences in diagnostic practices and outcomes in COSAB remain underexplored, particularly within the Australian healthcare setting.

This study aimed to address this gap by investigating whether sex influences diagnostic testing, clinical management, and 30-day mortality among adults with COSAB in Australia. Understanding these sex-based differences is essential for improving diagnostic accuracy, optimising treatment pathways, and ultimately reducing SAB-related mortality.

## Methods

This retrospective, single-centred study examined adult cases of COSAB diagnosed at University Hospital Geelong (UHG) between 2015 and 2020. UHG, a tertiary referral hospital in southwestern Victoria, Australia, serves a primary catchment area with a population of approximately 350,000, which increases to 500,000 for certain tertiary services extending to the South Australian border. Additionally, the hospital experiences around seven million visitors annually, further influencing service demand within Barwon Health [18]. Clinical and laboratory data were gathered from electronic medical records and managed using the Research Electronic Data Capture (REDCap) platform [19].

### Inclusion and exclusion

COSAB was defined as the isolation of a monomicrobial *S. aureus* blood culture within 48 h of hospital admission, while hospital-acquired SAB (HASAB) was classified as cases where the first positive blood culture was obtained ≥ 48 h after admission. This study included adult patients (≥ 18 years) with confirmed COSAB and excluded those with HASAB. To prevent duplicate inclusion in cases of recurrent bacteraemia, each patient was enrolled only once, based on the first positive blood culture obtained upon admission.

In addition, exclusion criteria included patients transferred to another hospital within the first seven days of admission, as follow-up data and key clinical information were inaccessible. Patients with polymicrobial bacteraemia involving *S. aureus* were also excluded.

### Data collection and variables

Data collected included demographics, clinical characteristics, microbiological data, and diagnostic investigations. Demographic variables included age, Aboriginal and Torres Strait Islander status, and the CACI score. Sex was defined as sex assigned at birth. Comorbidities included immunosuppression, the presence of an indwelling medical device (IMD), and being a person who injects drugs (PWID), defined as any injection of drugs within the six months preceding admission, based on the patient’s history.

Clinical characteristics encompassed self-reported symptoms such as fever, rigors, nausea/malaise, lethargy, and localised or general pain. The source of infection was classified based on the primary clinical presentation, including conditions like infective endocarditis (IE), osteomyelitis (OM), and sepsis. The portal of entry was categorised as skin and soft tissue infections (SSTI), diabetic foot ulcers (DFU), pneumonia, IMD associated infections, PWID associated infections, or unknown origin.

Diagnostic investigations were recorded in two timeframes: within the first seven days of admission and beyond seven days, as well as overall use. These timeframes provided a comprehensive view of early versus late-stage diagnostic workup, which could impact patient outcomes, particularly the development of complications like IE or other later-onset issues. The diagnostic tests included transoesophageal echocardiography (TOE), transthoracic echocardiography (TTE), x-ray, magnetic resonance imaging (MRI), computed tomography/positron emission tomography (CT/PET), and ultrasound (US).

Complications recorded in the electronic medical records included acute kidney injury (AKI), thrombus formation, aneurysm, encephalitis, IE, OM, septic arthritis, discitis/vertebral osteomyelitis, and sepsis. Microbiological data included susceptibility profiles denoted as Methicillin-sensitive *S. aureus* (MSSA), Methicillin-resistant *S. aureus* (MRSA), and Penicillin-sensitive *S. aureus* (PSSA), as reported on pathology reports.

### Clinical interventions and outcomes

The primary outcome of this study was 30-day all-cause mortality. Interventions such as (intensive care unit) ICU admission, surgical procedures, infectious diseases consultation (IDC), and the persistence of bacteraemia (positive blood cultures beyond three days) were assessed, along with appropriate antimicrobial therapy based on Australian guidelines for antibiotic activity against the identified isolate.

### Statistical analysis

An incidence-based approach was adopted for this retrospective study. This method involved capturing all eligible cases of COSAB occurring within the study’s defined time frame. Using a *post-hoc* power analysis using R statistical software v4.4.1 (R Core Team), the identified sample size of 279 with the proportions identified, power = 0.8 and significance = 0.05, there was an identified effect size of 0.34; suggesting the ability to identify small-to-medium effects.

Categorical data were summarised as counts and percentages (n, %), while continuous variables were reported as medians with interquartile ranges (IQRs), defined as the difference between the 75th and 25th percentiles [20]. Univariable analyses using binary logistic regression were conducted to (i) identify if sex was associated with 30-day mortality and (ii) assess if sex was associated with any of the clinical data. For continuous variables, independent-sample Mann Whitney U Test was performed to test for significant difference between the sexes. If found to be significant, the continuous variable would have been transformed into a binary cut-off and binary logistic regression was to occur, however this was not needed. Results were presented as odds ratios (ORs), 95% confidence intervals, and p-values less than 0.05 were considered statistically significant. Statistical analyses were performed using IBM SPSS Statistics version 29 (IBM Corporation, USA).

## Results

### Patient demographics and characteristics

A total of 279 cases COSAB were included in this study and 130 were excluded (11 not SAB, 54 hospital-acquired cases, 23 paediatric cases, 14 transferred, 28 polymicrobial). Among these, 58.4% (*n* = 163) were male, and 41.6% (*n* = 116) were female. All association data is presented in Table 1. The median age for males was 67 years (IQR: 29), while for females, it was 72 years (IQR: 28.75). The age distribution showed no significant difference between the sexes (*p* = 0.38), although distributed more to the right for females and more left for the males. The overall 30-day mortality rate was 19.0% (*n* = 53), with females exhibiting a higher mortality rate (21.6%) compared to males (17.2%), although this difference was not statistically significant (*p* = 0.36). For the entire cohort, the median age for fatal cases was 81 years (IQR: 16.0), significantly higher than for non-fatal cases, where the median age was 65 years (IQR: 27.3). The Charlson Age-Comorbidity Index (CACI) was significantly higher in fatal cases (median: 6, IQR: 4.0) compared to non-fatal cases (median: 4, IQR: 4). There was no significant difference between sex and CACI scores (*p* = 0.71).

**Table 1:** Univariable analysis of variables to identify associations with sex in COSAB

Variables	Total % (n) *n* = 279	Male % (*n*) *n* = 163	Female % (*n*) *n* = 116	Odds Ratio	95% CI for odds ratio	*p*-value
**Demographics**						
30-Day Mortality	19.0 (53)	17.2 (28)	21.6 (25)	1.33	(0.73-2.42)	0.36
Aboriginal and Torres Strait Islanders	1.4 (4)	1.2 (2)	1.7 (2)	1.41	(0.20-10.17)	0.73
Immunosuppression	19.4 (54)	22.1 (36)	15.5 (18)	0.65	(0.35–1.21)	0.17
Indwelling Medical Device	28.7 (80)	29.4 (48)	27.6 (32)	0.91	(0.54–1.55)	0.74
People Who Inject Drugs	13.6 (38)	11.7 (19)	16.4 (19)	1.49	(0.75–2.95)	0.26
**Symptoms**						
Fever	59.1 (165)	63.8 (104)	52.6 (61)	0.63	(0.39–1.02)	0.06
Rigors/Chills	18.3 (51)	20.9 (34)	14.7 (17)	0.65	(0.34–1.23)	0.19
Nausea/Malaise	12.9 (36)	9.8 (16)	17.2 (20)	1.02	(0.58–1.81)	0.95
Lethargy	41.6 (116)	39.5 (96)	55.6 (20)	1.91	(0.95–3.88)	0.07
Pain	62.4 (174)	60.7 (99)	64.7 (75)	1.18	(0.72–1.94)	0.51
**Source of Infection**						
Skin and soft tissue infection	27.6 (77)	28.2 (46)	26.7 (31)	0.93	(0.54–1.58)	0.78
Diabetic foot ulcer	5.7 (16)	6.1 (10)	5.2 (6)	0.84	(0.30–2.36)	0.73
Pneumonia	4.3 (12)	4.3 (7)	4.3 (5)	1.00	(0.31–3.25)	1.00
Indwelling Medical Device	11.8 (33)	11 (18)	12.9 (15)	1.20	(0.58–2.48)	0.61
Drug Injection site	4.7 (13)	4.9 (8)	4.3 (5)	0.87	(0.28–2.74)	0.82
Unknown	24 (67)	25.8 (42)	21.6 (25)	0.79	(0.45–1.39)	0.42
**Diagnostic Tests Yes/No**						
Transoesophageal Echocardiogram	**35.5 (99)**	**40.5 (66)**	**28.4 (33)**	**0.58**	**(0.35–0.97)**	**0.04**
Transthoracic Echocardiogram	74.9 (209)	74.8 (122)	75 (87)	1.01	(0.58–1.75)	0.98
X-Ray	94.3 (263)	93.9 (153)	94.8 (110)	1.20	(0.42–3.40)	0.73
Magnetic Resonance Imaging	30.5 (85)	30.1 (49)	31.0 (36)	1.05	(0.63–1.76)	0.86
Computed Tomography or Positron Emission Tomography	58.8 (164)	55.8 (91)	62.9 (73)	1.34	(0.83–2.19)	0.24
Ultrasound	30.8 (86)	32.5 (53)	28.4 (33)	0.83	(0.49–1.39)	0.47
**Diagnostic Tests (Performed in the first 7 days of admission)**						
Transoesophageal Echocardiogram	17.2 (48)	19.6 (32)	13.8 (16)	0.66	(0.34–1.26)	0.21
Transthoracic Echocardiogram	65.2 (182)	65.6 (107)	64.7 (75)	0.96	(0.58–1.58)	0.86
X-Ray	88.9 (248)	89.0 (145)	88.8 (103)	0.98	(0.46–2.10)	0.97
Magnetic Resonance Imaging	24.0 (67)	23.3 (38)	25.0 (29)	1.10	(0.63–1.91)	0.75
Computed Tomography or Positron Emission Tomography	51.6 (144)	50.3 (82)	53.4 (62)	1.13	(0.70–1.83)	0.61
Ultrasound	25.8 (72)	27.6 (45)	23.3 (27)	0.80	(0.46–1.38)	0.42
**Diagnostic test (Performed in post 7 days of admission)**						
Transoesophageal Echocardiogram	20.4 (57)	23.9 (39)	15.5 (18)	0.59	(0.32–1.08)	0.09
Transthoracic Echocardiogram	15.4 (43)	16.6 (27)	13.8 (16)	0.81	(0.41–1.58)	0.53
X-Ray	41.2 (115)	40.5 (66)	42.2 (49)	1.08	(0.66–1.74)	0.77
Magnetic Resonance Imaging	11.5 (32)	12.3 (20)	10.3 (12)	0.83	(0.39–1.76)	0.62
Computed Tomography or Positron Emission Tomography	16.8 (47)	14.7 (24)	19.8 (23)	1.43	(0.76–2.69)	0.26
Ultrasound	9.3 (26)	9.2 (15)	9.5 (11)	1.03	(0.46–2.34)	0.94
**Complications**						
Acute Kidney Injury	31.5 (88)	28.2 (46)	36.2 (42)	1.44	(0.87–2.40)	0.16
Thrombus	8.6 (24)	6.1 (10)	12.1 (14)	2.10	(0.90–4.91)	0.09
Aneurysm*	2.5 (7)	2.5 (4)	2.6 (3)	1.06	(0.23–4.82)	0.94
Encephalitis	1.1 (3)	1.2 (2)	0.9 (1)	0.70	(0.06–7.81)	0.77
Infective Endocarditis	20.1 (56)	18.4 (30)	22.4 (26)	1.28	(0.71–2.31)	0.41
Osteomyelitis	15.4 (43)	17.8 (29)	12.1 (14)	0.63	(0.32–1.26)	0.19
Septic Arthritis	12.5 (35)	9.8 (16)	16.4 (19)	1.80	(0.88–3.67)	0.11
Discitis/Vertebral Osteomyelitis	5.0 (14)	6.1 (10)	3.4 (4)	0.55	(0.17–1.79)	0.32
Septicaemia	39.4 (110)	41.7 (68)	36.2 (42)	0.79	(0.49–1.30)	0.35
**Microbiological variables**						
PSSA	12.5 (35)	15.3 (25)	8.6 (10)	0.52	(0.24–1.13)	0.10
MSSA	**79.6 (222)**	**74.8 (122)**	**86.2 (100)**	**2.10**	**(1.13–3.97)**	**0.02**
MRSA	7.9 (22)	9.8 (16)	5.2 (6)	0.50	(0.19–1.32)	0.16
**Interventions (other)**						
ICU admission	28.0 (78)	28.8 (47)	26.7 (31)	0.90	(0.53–1.53)	0.70
Surgery	28.2 (78)	28.6 (46)	27.6 (32)	0.95	(0.56–1.62)	0.86
Infectious Diseases Consult**	95.7 (266)	94.4 (153)	97.4 (113)	2.22	(0.59–8.37)	0.24
Persistence	19.0 (53)	17.2 (28)	21.6 (25)	1.33	(0.73–2.42)	0.36
Appropriate antimicrobial Treatment	98.9 (276)	98.8 (161)	99.1 (115)	1.43	(0.13–15.94)	0.77
Palliation	18.3 (51)	18.4 (30)	18.1 (21)	0.98	(0.53–1.82)	0.95

MSSA = Methicillin-Susceptible *Staphylococcus aureus*, PSSA = Penicillin-Susceptible Staphylococcus aureus, MRSA = Methicillin-Resistant *Staphylococcus aureus*, ICU = Intensive Care Unit. OR=Odds Ratio, 95% CI = 95% Confidence Interval. Male = 0, Female = 1 for statistical coding. Statistical significance was set at *p* < 0.05. * Aneurysm missing 2 data points, ** Infectious Disease consultation missing 1 data point

### Self-reported symptoms at admission

Males more frequently reported fever (63.8% vs. 52.6%, *p* = 0.06), rigors (20.9% vs. 14.7%, *p* = 0.19), but reported pain less frequently (60.7% vs. 64.7%, *p* = 0.51) compared to females. In contrast, lethargy was more commonly reported by females (17.2%) than males (9.8%), but this was not statistically associated (*p* = 0.07).

### Infection sources

The source of infection was unknown in 24.0% of cases, with a slightly higher proportion in males (25.8%) compared to females (21.6%). Skin and soft tissue infections (SSTIs) were the most common infection source for both sexes, with no significant difference observed (males: 28.2%, females: 26.7%, *p* = 0.78).

### Diagnostic testing

Females underwent transoesophageal echocardiography (TOE) significantly less frequently to males (28.4% compared to 40.5%; OR 0.58, 95% CI: 0.35–0.97, *p* = 0.04). On investigation to see if TOE rates differed in the first seven days, it was observed that females were underwent TOE less frequently in the first seven days of admission compared to males (13.8% vs. 19.6%), however was not statistically associated (*p* = 0.21). In contrast, the use of transthoracic echocardiography (TTE) was similar between sexes (*p* = 0.98).

### Microbiological and clinical presentations

Females were more often infected with methicillin-susceptible *S. aureus* (MSSA) infections (OR 2.10, 95% CI: 1.11–3.97, *p* = 0.02), while the incidence of methicillin-resistant *S. aureus* (MRSA) did not differ significantly between sexes. Complications such as infective endocarditis (IE), septic arthritis, thrombus, and acute kidney injury were not significantly associated with sex (IE: *p* = 0.41, septic arthritis: *p* = 0.11, thrombus: *p* = 0.09, acute kidney injury: *p* = 0.16).

### Clinical management

Males had a longer median hospital stay (26 days, IQR: 33.25 compared to females (18 days, IQR: 34, *p* = 0.51). Conversely, fatal cases had a shorter median hospital stay (8.0 days, IQR: 7.0) compared to non-fatal cases (29 days, IQR: 32). ICU admission rates were higher in fatal cases (37.7% vs. 25.7%) but were similar between males (28.8%) and females (26.7%), with no significant association (*p* = 0.70). Surgical interventions were performed in 28.6% of males and 27.6% of females, with no significant association (OR 0.95, 95% CI: 0.56–1.62, *p* = 0.86). Palliative care was administered to 18.1% of females and 18.4% of males, with 79.2% of fatal cases receiving palliative care compared to just 4.0% of non-fatal cases.

## Discussion

Although the literature remains conflicted, there is strengthening evidence that sex has an influence on SAB mortality [11, 12, 21, 22]. Importantly, the underlying mechanisms for this influence has not been explored in depth. In our study, the 30-day all-cause mortality rate was 19.0%, exceeding the reported national averages of 17.5% for all SAB cases and 17.0% for COSAB in Australia in 2022 [4]. Several factors may explain this discrepancy, such as temporal effects over the 6-year collection period, as well as the study’s setting in a large tertiary referral hospital that routinely manages more complex and critically ill patients, often transferred from smaller healthcare facilities.

Our study included a cohort of 58.4% males and 41.6% females, consistent with the documented male predominance in SAB incidence [9, 10, 23]. While some studies have reported higher mortality in females with bloodstream infections [6, 20, 21], our study found no significant association between female sex and 30-day all-cause mortality in COSAB, with mortality rates of 21.6% in females and 17.2% in males (*p* = 0.36). Despite this, these findings do not exclude the potential for a sex-based effect. Females were older than males (males median age: 67 years (IQR: 29), female median age: 72 years (IQR: 28.75)), which may have explained the difference in mortality between males and females. The limited sample size reduced the statistical power to detect smaller but clinically meaningful differences and made assessing for interactions and adjusted analyses not possible.

Importantly, we found that while post-diagnosis antimicrobial treatment was similar between the sexes, differences in diagnostic practices may exist. In this study, we observed a notable sex-based disparity in the overall use of transoesophageal echocardiography (TOE), with females undergoing the procedure significantly less frequently than males (OR: 0.58, 95% CI: 0.35–0.97, *p* = 0.04), despite no association between infective endocarditis (IE) and sex (OR: 1.28, 95% CI: 0.71–2.31, *p* = 0.41) or persistent blood cultures (OR: 1.33, 95% CI: 0.73–2.42, *p* = 0.36). This lower use of TOE in females may contribute to delayed or missed diagnoses, potentially impacting clinical outcomes [12] or possibly reflect different presentations changing diagnostis practices. These findings reflect broader concerns about sex-based disparities in medical decision-making and diagnostic evaluation. Notably, similar patterns have been reported in other studies, with evidence suggesting lower use of TOE and other diagnostic modalities in females compared to males [9, 11, 24, 25].

Sex-based differences in diagnostic testing and clinical management extend beyond infections and have been documented in other clinical areas, such as cardiology. Studies have shown that lower intervention rates in females have been partly attributed to inadequate monitoring and under recognition of cardiovascular risk in females, as well as the more variable and heterogeneous clinical presentations that can obscure timely diagnosis [26, 27]. For example, Poisson regression models have demonstrated that females are less likely than males to receive key cardiovascular disease interventions such as lipid-lowering medications, antihypertensives, and, importantly, invasive cardiac procedures, despite having similar clinical indications [27]. While some of these interventions are diagnostic, such as coronary angiography, others are therapeutic and critical for managing disease progression.

This discrepancy may reflect systemic factors, including physician risk perception, where implicit biases lead clinicians to associate certain conditions with one sex over the other [12]. In the case of infective endocarditis, such biases may contribute to a lower referral rate for advanced imaging such as transoesophageal echocardiography (TOE) in females, based on the perception that the condition is more common in males [28]. Additionally, sex-based differences in the clinical presentation of endocarditis may compound these biases. For instance, females may present with more atypical symptoms, reducing clinical suspicion and delaying diagnostic imaging such as TOE [12]. These delays are critical, as early intervention is needed for improving outcomes in endocarditis [26].

While diagnostic disparities may account for some of the differences in outcomes, underlying biological factors are also likely to influence how infections manifest and progress in males and females. Hormonal influences and comorbidities have been proposed as contributing factors that shape immune responses and the clinical presentation of infections [29]. For instance, hormonal fluctuations in females may impact immune function and contribute to delayed or missed diagnoses [29]. However, this was outside the scope of our study and so further research is warranted.

Our study found females were significantly more frequently found to have methicillin-sensitive *S. aureus* (MSSA) infections than males (OR 2.10, 95% CI 1.13–3.97, *p* = 0.02). Methicillin-resistant *S. aureus* (MRSA) strains, represent about 12.4% of community-acquired cases in bacteraemia in Australia [30] and in Victoria, this study’s setting, represent 10.8% of cases [30], aligning with this study’s findings. Approximately 45.8% of these national MRSA strains harbour the Panton-Valentine Leukocidin genes [30]. Although MSSA is generally associated with less severe outcomes than methicillin-resistant *S. aureus* (MRSA) [15], the implications of this sex-based difference in antibiotic susceptibility type remain unclear. It may reflect variations in host–pathogen interactions, healthcare exposure, or infection sources; however, in our cohort, the source of infection did not differ significantly between sexes. It should be noted that colonisation status is not routinely assessed at the site hospital, so could be a potential explanation for these differences. Further research is needed to clarify the biological and clinical significance of these findings.

Analysis of treatment and interventions showed no sex-based differences in antimicrobial therapy, with 99% of males and females receiving appropriate treatment. This finding supports existing literature, which indicates that antibiotic stewardship practices are typically sex-neutral across healthcare settings [2, 11]. However, it’s important to note we did not look at differences in duration or specific choice of antibiotics due to the group stratification that would be involved, which would have required a larger sample size. Similarly, no significant differences were found in surgical interventions, with an odds ratio of 0.95 (95% CI: 0.56–1.62, *p* = 0.86), suggesting that surgical management was comparable between sexes. The likelihood of receiving an infectious diseases consult also showed no significant difference (OR 2.22, 95% CI: 0.59–8.37, *p* = 0.24, 1 missing data point), aligning with prior studies indicating no sex-based disparities in IDC referrals [11, 31, 32]. These findings suggest that both sexes receive similar care in these areas.

### Limitations and future directions

While this study provides valuable insights into sex-based disparities, it is not without limitations. The retrospective, single-centre design, conducted within an Australian setting, limits the generalisability of findings to other populations, particularly those with differing healthcare systems or demographic characteristics and introduces potential biases that often occur with retrospective studies.

The modest sample size may also have reduced the ability to detect smaller, yet clinically meaningful, differences in outcomes between sexes as well as limit our ability to stratify or assess for meaningful interactions. Larger, multi-centre studies across diverse healthcare environments are needed to validate these findings. Further investigation into the impact of diagnostic disparities, such as the lower use of transoesophageal echocardiography (TOE) in females, remains essential for understanding sex-based differences in clinical outcomes.

### Novelty of the study

The novelty of this study lies in its examination of sex-based disparities in the clinical management of COSAB, an area that remains underexplored in current literature. While sex-based differences in SAB management have been recognised, this study specifically highlights the relative lower use of transoesophageal echocardiography (TOE) in female patients, offering new insights into potential diagnostic and management biases that may contribute to poorer clinical outcomes.

## Conclusion

This study underscores the importance of clinical decision-making in COSAB and highlights an unrecognised use of sex as a considered factor in the risk stratification process which does not currently add value to clinical outcome. Although no significant differences in 30-day mortality were found between sexes, the findings highlight areas where healthcare practices could be improved to ensure equitable care for both males and females. Specifically, the lower use of TOE in females points to a gap in diagnostic practices that may negatively impact patient outcomes. Further research into the causes of these disparities, including the impact of diagnostic and clinical management differences, is essential to inform better clinical guidelines and ultimately improve outcomes for all patients with COSAB.

## Data Availability

The datasets used and/or analysed during the current study are available from the corresponding author on reasonable request.

## Abbreviations

BHHREC: Barwon Health Human Research Ethics Committee
CACI: Charlson Comorbidity Weighted Index
COSAB: Community-Onset *Staphylococcus aureus* Bacteraemia
CI: Confidence Interval
CT/PET: Computed Tomography / Positron Emission Tomography
DFU: Diabetic Foot Ulcer
DUHREC: Deakin University Human Research Ethics Committee
HASAB: Healthcare-Associated *Staphylococcus aureus* Bacteraemia
ICU: Intensive Care Unit
IDC: Infectious Disease Consultation
IE: Infective Endocarditis
IMD: Indwelling Medical Device
IQR: Inter-Quartile Range
MRI: Magnetic Resonance Imaging
MRSA: Methicillin-Resistant *Staphylococcus aureus*
MSSA: Methicillin-Sensitive *Staphylococcus aureus*
OR: Odds Ratio
PSSA: Penicillin-Sensitive *Staphylococcus aureus*
PWID: Person who injects drugs
*S. aureus*: *Staphylococcus aureus*
SAB: *Staphylococcus aureus* Bacteraemia
SSTI: Skin and Soft Tissue Infection
TOE: Transesophageal Echocardiography
TTE: Transthoracic Echocardiography
UHG: University Hospital Geelong
